# New York Therapeutical Society

**Published:** 1878-03

**Authors:** 


### NEW YORK THERAPEUTICAL SOCIETY.

NEW YORK, February 8, 1878.

Dr. James R. Leaming, President, in the chair.

Dr. E. C. Seguin, Chairman of the Committe on Neurotics, made the report, of which the following is a brief summary. The experiments in the first instance were made by noting the number of epileptic attacks occuring in a certain number of patients during a certain period of time.

*First.*—Noting the number of attacks which occurred in a certain number of patients while receiving a placeboic treatment, during a certain period of time, and also the number of attacks occurring in the same patients while under the bromine treatment for a corresponding length of time.

*Second.*—Noting the number of attacks occurring while under the chloride of potassium treatment, and comparing it with the number of attacks occurring in the same patients during treatment by bromides for a corresponding length of time.

The following were among the conclusions reached:

1. That bromide of potassium had a positive restraining influence over epilepsy, both in reducing the number and the severity of the epileptic attacks.

2. That chloride of potassium was almost inert as an agent in the treatment of epilepsy.

It was inferred that the potassium could not be the efficient element in controlling the epileptic attacks, because the chloride of potassium contained more potassium than did the bromide of potassium.

The formula employed by Dr. Seguin and two of his clinical assistants was as follows:

℞.—Potas. bromid, . . . . . . ℥i.

Chloral hydrate, . . . . . . ℥ss.

Aquæ . . . . . . . . ℥vii.

M.

Of that mixture, from four to six teaspoonfuls were administered daily.

The observations were made by Dr. A. McLane Hamilton, of New York; Dr. J. C. Shaw, of Brooklyn; Dr. E. C. Seguin and two of his clinical assistants, Drs. T. A. McBride and N. B. Emerson. The combination of chloral with bromide of potassium had been used in twenty-eight cases, and it was shown that the epileptic attacks were warded off by the new solution quite as well as by the bromides alone (bromide of ammonium in the same quantity being used instead of the chloral hydrate). The reflex irritability of the throat was reduced equally as well as by the use of the bromides alone.

In all the cases there was a remarkable immunity from the bad effects of the bromides, especially in the psychic sphere, and little or no ache has been produced. In no case was more than forty-five grains of each drug given daily, and really no bad results had been realized under the new treatment.

It had been hoped that the admixture of the chloral with the bromide would diminish the evil results produced by the bromide, such as general depression of the system, irritation of the skin, etc., and those advantages had been partially, if not fully, realized. The good results obtained had encouraged the committee to make more extensive trial of the new mixture.

Dr. W. H. Thompson thought it quite probable that the apparent good effect obtained by the use of chlorals as indicated would prove but temporary.

He always used phosphorus and cod-liver oil with the bromides in the treatment of epilepsy, relying upon the phosphorus to prevent the bromic acne.

Dr. E. R. Squibb remarked that he had not yet found any one who had seen an attack of *grand mal* occur when the patient, at the time of its occurrence, was suffering from bromism. The history of four cases of epilepsy was referred to, which had been treated by the use of bromide of potassium alone. The manner of administration had been to give the drug in gradually increasing doses until the attacks were arrested. Little or nothing had been seen of the bad effects produced by the bromides. Intermissions in the use of the bromide had been given the patient, varying in length from two to ten days, and to these a good deal of the absence of the bad effects of the remedy was attributed.

Dr. F. A. Castle remarked that he had employed chloral and bromide of potassium in combination in the treatment of other conditions than the epileptic, and had found that a less quantity was required than would have been necessary had either drug been used alone.

Dr. E. C. Seguin remarked that his experience was against the omission of the bromide for even a single dose in the treatment of epilepsy.

Dr. Thomson endorsed Dr. Seguin's experience in that particular. He also referred to impressions on the surface nerves as a means for diminishing the quantity of bromide required to control the epileptic attacks. It was his habit, therefore, to produce surface irritation on epileptic patients by the use of a drachm of red pepper to a pint or quart of water, and applying it with a sponge.

Dr. H. T. Hanks remarked that although the effect of the chloral combined with the bromides in the treatment of epilepsy might be merely palliative, yet in that way it assisted nature in affecting a cure of the disease, or gave other remedies an opportunity to operate.

Dr. Squibb referred to the use of nitrite of amyl for the same purpose. It was a remedy which produced effects much more evanescent than those produced by chloral, yet it had been successfully used for preventing the occurrence of epileptic attacks. Although not directly curative, it had proved in many cases a valuable adjuvant in the treatment of the disease, because it obviated the retarding influence produced by a convulsion.

Dr. H. G. Piffard exhibited a proposed new Dover's powder, made by substituting sugar of milk for sulphate of potash in the old formula. He also exhibited pilocarpin, put up in a convenient form for triturating the alkaloid with one hundred parts of sugar of milk.

A specimen of Calcutta flaxseed was also exhibited, which had been suggested as a substitute for cod-liver oil.

Dr. E. C. Seguin objected to the use of the term "trituration," because of its immediate association with a fraudulent school in medicine.

After a brief discussion on this point, the society adjourned.

